# Handwashing and disinfection precautions taken by U.S. adults to prevent coronavirus disease 2019, Spring 2020

**DOI:** 10.1186/s13104-020-05398-3

**Published:** 2020-12-04

**Authors:** Laura G. Brown, E. Rickamer Hoover, Catherine E. Barrett, Kayla L. Vanden Esschert, Sarah A. Collier, Amanda G. Garcia-Williams

**Affiliations:** 1 Centers of Disease Control and Prevention, COVID-19 Response, 1600 Clifton Road, Atlanta, GA USA; 2 Centers for Disease Control and Prevention, National Center for Environmental Health, 4770 Buford Highway, Atlanta, GA USA

**Keywords:** COVID-19, Coronavirus, Handwashing, Surface disinfection, Demographic differences

## Abstract

**Objectives:**

The objectives of this study were to assess self-reported hygiene precautions taken by U.S. adults during spring 2020 to prevent coronavirus disease 2019 (COVID-19) and to identify demographic characteristics associated with these hygiene precautions.

**Results:**

We obtained data from Porter Novelli Public Services’s national survey, *Spring ConsumerStyles*, conducted March 19–April 9, 2020 among a nationally representative random sample of 6463 U.S. adults aged 18 years or older. We present data from the survey question: “What, if any, precautions are you taking to prevent coronavirus?”. Respondents replied yes or no to the following precautions: washing hands often with soap and water and disinfecting surfaces at home and work often. Most respondents reported taking hygiene-related precautions to prevent COVID-19; more respondents reported handwashing (93%) than disinfecting surfaces (74%). Men, younger respondents, those with lower income and education levels, and respondents in self-rated poor health had lower reported rates of both handwashing and disinfecting surfaces. Communications about hygiene precautions for COVID-19 prevention may need to target sub-populations with the greatest gaps in hygiene-related practices. Research identifying barriers to these practices and developing effective messaging could inform and improve these communications.

## Introduction

From January through October 2020, over 9 million cases of coronavirus disease 2019 (COVID-19), caused by the virus SARS-CoV-2, were reported in the U.S.; these included 229,978 deaths [1]. Although SARS-CoV-2 is thought to spread mainly from person to person, it is possible that people can become infected by touching something with the virus on it and then touching their own mouth, nose, or possibly eyes [2]. CDC has provided guidance to the public to help prevent the spread of COVID-19, including handwashing and cleaning and disinfection guidance [3]. These recommendations include handwashing with soap and water for at least 20 s, using hand sanitizer with at least 60% alcohol, and cleaning and disinfecting frequently touched surfaces daily, with products approved by the Environmental Protection Agency. To assess the U.S. public’s adherence to these recommendations, we analyzed national survey data on respondents’ self-reported hygiene-related precautions (handwashing and surface disinfection) taken to prevent COVID-19. We also examined associations between respondents’ demographic characteristics and their self-reported hygiene-related precautions.

## Main text

### Methods

#### Participants and sampling

Porter Novelli Public Services administers a national survey, called *Spring ConsumerStyles,* conducted using Ipsos’ KnowledgePanel, which is representative of the non-institutionalized U.S. population [4]. Respondents are randomly recruited by mail through probability-based, stratified sampling to participate in the survey panel. They receive the surveys through email and complete them online. If needed, respondents were provided with a laptop or tablet and access to the Internet, to help ensure that those with technological limitations were not excluded from the survey. Respondents receive reward points they can redeem online for gift cards or prizes. The 2020 *Spring ConsumerStyles* survey was conducted from March 19–April 9, 2020 and was sent via email to 11,097 participants. The final sample included 6463 adults who completed the survey, yielding a response rate of 58.2%, which was similar to the 2019 *Spring ConsumerStyles* survey [4]. The analyses were weighted to match the U.S. Census 2019 U.S. Current Population Survey proportions by gender, age, annual household income, race/ethnicity, household size, education, census region, metropolitan status, and parental status. See the Porter Novelli website for more information on *ConsumerStyles* methodology [4].

#### Outcome variable

Coronavirus was defined for respondents as ‘the 2019 novel coronavirus’ as part of the survey. To assess hygiene-related precautions taken in response to COVID-19, respondents were asked, “What, if any, precautions are you taking to prevent coronavirus?”. They then responded yes or no to a series of actions, listed randomly. We present data on the following precautions: washing hands often with soap and water and disinfecting surfaces at home/work often (hereafter, referred to as handwashing and disinfecting surfaces).

#### Demographic variables

Data on demographic characteristics of gender, age, income, education, race/ethnicity, metropolitan status (metropolitan vs. non-metropolitan), and geographic region (based on U.S. Census regions) were collected when respondents agreed to participate in the panel; data on self-rated health were collected in the survey itself.

#### Analysis

We calculated percentages for both precautions, overall and by demographic characteristics (Table 1). We calculated prevalence ratios for comparisons across demographic categories by calculating the ratio of the prevalence of behavior in one demographic category compared to another (e.g., handwashing prevalence for females vs. males). Because we were interested in all comparisons across categories within characteristics (e.g., ages 18–29 vs. 30–44 vs. 45–59), we used multiple reference levels per characteristic. Prevalence ratios, 95% confidence intervals, and p-values were obtained using Zou’s modified Poisson approach [5] (Table 2). We used this modified approach because it provides the most accurate confidence intervals and p-values. Comparisons significant at p < .05 are discussed below. Multiple comparison adjustments were not made. We weighted the data to adjust for selection and nonresponse.

**Table 1:** Percentages of respondents who reported handwashing and disinfecting surfaces often to prevent coronavirus, by demographic characteristics – *Spring ConsumerStyles* 2020

Characteristic	Categories	Washing hands often n (weighted %)	Disinfecting surfaces often n (weighted %)
All respondents	–	6013 (93.4%)	4768 (74.0%)
Gender	Female	3153 (94.5)	2624 (78.7)
	Male	2860 (92.1)	2145 (69.1)
Age	18–29	1194 (91.4)	909 (69.6)
	30–44	1546 (90.1)	1182 (73.4)
	45–59	1546 (94.7)	1261 (77.2)
	≥ 60	1822 (96.4)	1417 (74.9)
Income	< $25k	745 (86.4)	587 (68.1)
	$25k–$49k	1050 (92.9)	850 (75.2)
	$50k–$99k	1977 (93.6)	1560 (73.9)
	≥ $100k	2242 (95.9)	1771 (75.8)
Education	< High school	550 (84.7)	463 (71.4)
	High school	1662 (91.7)	1324 (73.1)
	Some college	1713 (95.0)	1812 (76.6)
	≥ Bachelor’s degree	2088 (96.0)	1599 (73.5)
Race/Ethnicity	Non-Hispanic White	3847 (94.0)	2982 (72.8)
	Non-Hispanic Black	683 (91.2)	596 (79.6)
	Hispanic	964 (92.0)	816 (77.9)
	Multiracial/other	91 (96.4)	68 (72.7)
Self-rated health^a^	Excellent	2333 (95.4)	1874 (76.6)
	Very good	2237 (94.1)	1759 (74.0)
	Good	749 (90.0)	566 (68.0)
	Fair	577 (92.4)	470 (75.3)
	Poor	113 (75.2)	96 (63.4)
Region^b^	Northeast	1075 (95.4)	865 (76.8)
	Midwest	1248 (93.8)	994 (74.7)
	South	2264 (92.8)	1815 (74.4)
	West	1427 (92.5)	1094 (70.9)
Metropolitan status^c^	Metro	5233 (93.7)	4169 (74.6)
	Non-metro	780 (91.3)	560 (70.2)

^a^Health status assessed by asking “In general, would you say your health is…”

^b^Region categories based on U.S. Census

^c^Metropolitan status was defined as a core-based statistical area

**Table 2:** Prevalence ratios, confidence intervals, and p-values for comparisons of respondents’ self-reported handwashing and surface disinfecting, by demographic characteristics–*Spring ConsumerStyles* 2020

Characteristic	Comparison^a^	Washing hands often			Disinfecting surfaces often		
		% vs. %^a^	PR (95% CI)	p	% vs. %^a^	95% CI	p
Gender	Female vs. Male	*94.5* vs. *92.1*	*1.03 (1.01, 1.04)*	*.004*	*78.7* vs. *69.1*	*1.14 (1.10, 1.18)*	*.001*
Age	30–44 vs. 18–29	90.1 vs. 91.4	0.98 (0.95, 1.01)	.392	73.4 vs. 69.6	1.05 (0.98, 1.13)	.132
	45–59 vs. 18–29	*94.7* vs. *91.4*	*1.04 (1.01, 1.07)*	*.022*	*77.2* vs. *69.6*	*1.09 (1.00, 1.19)*	*.048*
	≥ 60 vs. 18–29	*96.4* vs. *91.4*	*1.05 (1.02, 1.09)*	*.001*	*74.9* vs. *69.6*	*1.08 (1.01, 1.15)*	*.026*
	45–59 vs. 30–44	*94.7* vs. *90.1*	*1.05 (1.03, 1.08)*	*.001*	*77.2* vs. *73.4*	*1.05 (1.01, 1.10)*	*.023*
	≥ 60 vs. 30–44	*96.4* vs. *90.1*	*1.07 (1.05, 1.10)*	*.001*	74.9 vs. 73.4	1.05 (0.99, 1.12)	.121
	≥ 60 vs. 45–59	*96.4* vs. *94.7*	*1.02 (1.00, 1.03)*	*.037*	74.9 vs. 77.2	0.97 (0.933, 1.01)	.125
Income	$25k–$49k vs. < $25k	*92.9* vs. *86.4*	*1.08 (1.03, 1.12)*	*.001*	*75.2* vs. *68.1*	*1.10 (1.02, 1.19)*	*.010*
	$50k–$99k vs. < $25k	*93.6* vs. *86.4*	*1.08 (1.04, 1.13)*	*.001*	*73.9* vs. *68.1*	*1.12 (1.01, 1.23)*	*.026*
	≥ $100k vs. < $25k	*95.9* vs. *86.4*	*1.11 (1.07, 1.16)*	*.001*	*75.8* vs. *68.1*	*1.11 (1.04, 1.19)*	*.002*
	$50k–$99k vs. $25k–$49k	93.6 vs. 92.9	1.01 (0.98, 1.03)	.520	73.9 vs. 75.2	0.98 (0.93, 1.04)	.508
	≥ $100k vs. $25k–$49k	*95.9* vs. *92.9*	*1.03 (1.00, 1.06)*	*.005*	75.8 vs. 75.2	1.00 (0.93, 1.07)	.984
	≥ $100k vs. $50k–$99k	*95.9* vs. *93.6*	*1.02 (1.01, 1.04)*	*.010*	75.8 vs. 73.9	1.03 (0.98, 1.07)	.242
Education	High school vs. < High school	*91.7* vs. *84.7*	*1.08 (1.02, 1.15)*	*.006*	73.1 vs. 71.4	1.02 (0.94, 1.12)	.595
	Some college vs. < High school	*95.0* vs. *84.7*	*1.12 (1.06, 1.19)*	*.001*	76.6 vs. 71.4	1.11 (0.99, 1.24)	.074
	≥ Bachelor’s degree vs. < High school	*96.0* vs. *84.7*	*1.13 (1.07, 1.20)*	*.001*	73.5 vs. 71.4	1.03 (0.95, 1.12)	.494
	Some college vs. High school	*95.0* vs. *91.7*	*1.04 (1.01, 1.06)*	*.002*	76.6 vs. 73.1	1.05 (1.00, 1.10)	.054
	≥ Bachelor’s degree vs. High school	*96.0* vs. *91.7*	*1.04 (1.02, 1.07)*	*.001*	73.5 vs. 73.1	1.02 (0.96, 1.09)	.535
	≥ Bachelor’s degree vs. Some college	96.0 vs. 95.0	1.01 (0.99, 1.03)	.210	*73.5* vs. *76.6*	*0.96 (0.92, 1.00)*	*.043*
Race/Ethnicity	Non-Hispanic Black vs. Non-Hispanic White	91.2 vs. 94.0	0.97 (0.94, 1.00)	.080	*79.6* vs. *72.8*	*1.09 (1.04, 1.15)*	*.001*
	Hispanic vs. Non-Hispanic White	92.0 vs. 94.0	0.98 (0.95, 1.01)	.141	*77.9* vs. *72.8*	*1.07 (1.02, 1.13)*	*.011*
	Multiracial/Other vs. Non-Hispanic White	94.5 vs. 94.0	1.01 (0.97, 1.04)	.752	68.1 vs. 72.8	0.94 (0.86, 1.02)	.120
	Hispanic vs. Non-Hispanic Black	92.0 vs. 91.2	1.01 (0.97, 1.05)	.679	77.9 vs. 79.6	0.98 (0.91, 1.05)	.531
	Multiracial/Other vs. Non-Hispanic Black	94.5 vs. 91.2	1.04 (0.99, 1.08)	.117	*68.1* vs. *79.6*	*0.85 (0.78, 0.94)*	*.001*
	Multiracial/Other vs. Hispanic	94.5 vs. 92.0	1.03 (0.99, 1.07)	.198	*68.1* vs. *77.9*	*0.88 (0.80, 0.96)*	*.005*
Self-rated health	Fair vs. Poor	*90.0* vs. *75.2*	*1.20 (1.05, 1.36)*	*.007*	68.0 vs. 63.4	1.07 (0.90, 1.27)	.418
	Good vs. Poor	*94.1* vs. *75.2*	*1.25 (1.10. 1.42)*	*.001*	74.0 vs. 63.4	1.17 (0.99, 1.37)	.061
	Very good vs. Poor	*95.4* vs. *75.2*	*1.27 (1.12, 1.44)*	*.001*	*76.6* vs. *63.4*	*1.21 (1.03, 1.42)*	*.022*
	Excellent vs. Poor	*92.4* vs. *75.2*	*1.22 (1.08, 1.40)*	*.002*	*75.3* vs. *63.4*	*1.19 (1.00, 1.41)*	*.046*
	Good vs. Fair	*94.1* vs. *90.0*	*1.05 (1.01, 1.08)*	*.011*	*74.0* vs. *68.0*	*1.09 (1.02, 1.16)*	*.016*
	Very good vs. Fair	*95.4* vs. *90.0*	*1.06 (1.02, 1.10)*	*.001*	*76.6* vs. *68.0*	*1.13 (1.05, 1.20)*	*.001*
	Excellent vs. Fair	92.4 vs. 90.0	1.03 (0.98, 1.07)	.236	*75.3* vs. *68.0*	*1.11 (1.02, 1.20)*	*.018*
	Very good vs. Good	95.4 vs. 94.1	1.01 (1.00, 1.03)	.134	76.6 vs. 74.0	1.04 (1.00, 1.08)	.084
	Excellent vs. Good	92.4 vs. 94.1	0.98 (0.95, 1.01)	.273	75.3 vs. 74.0	1.02 (0.96, 1.09)	.590
	Excellent vs. Very good	92.4 vs. 95.4	0.97 (0.94, 1.00)	.051	75.3 vs. 76.6	0.98 (0.92, 1.05)	.596
Region	Northeast vs. Midwest	95.4 vs. 93.8	1.02 (1.00, 1.04)	.128	76.8 vs. 74.7	1.03 (0.98, 1.08)	.301
	West vs. Midwest	92.5 vs. 93.8	0.99 (0.96, 1.01)	.265	70.9 vs. 74.7	0.95 (0.90, 1.00)	.058
	West vs. South	92.5 vs. 92.8	1.00 (0.97, 1.02)	.788	70.9 vs. 74.4	0.95 (0.91, 1.00)	.065
	Midwest vs. South	93.8 vs. 92.8	1.01 (1.00, 1.03)	.350	74.7 vs. 74.4	1.00 (0.96, 1.05)	.843
	Northeast vs. South	*95.4* vs. *92.8*	*1.03 (1.01, 1.05)*	*.013*	76.8 vs. 74.4	1.03 (0.98, 1.09)	.196
	Northeast vs. West	*95.4* vs. *92.5*	*1.03 (1.01, 1.06)*	*.012*	*76.8* vs. *70.9*	*1.08 (1.02, 1.15)*	*.005*
Metropolitan status	Metro vs. Non-metro	93.7 vs. 91.3	1.03 (1.00, 1.06)	.082	*74.6* vs. *70.2*	*1.06 (1.00, 1.13)*	*.037*

PR: Prevalence ratio; CI: confidence interval

^a^The second category or number listed is the reference level for the prevalence ratio

Italics indicates significant comparisons (p < .05)

### Results

#### Handwashing (Tables 1 and 2)

Almost all (93.4%) respondents said they were washing their hands often with soap and water to prevent COVID-19. Women more often reported frequent handwashing than men. In general, respondents in older age categories more often reported frequent handwashing than respondents in younger age categories. Respondents 60 years of age or older more often reported frequent handwashing than respondents in all younger age categories (18–29 year-olds, 30–44 year-olds, and 45–59 year-olds). Respondents with higher incomes more often reported frequent handwashing than respondents with lower incomes, with the exception of one comparison ($50k–$99k vs. $25k–$49k). Respondents with higher educational levels more often reported frequent handwashing than respondents with lower educational levels, with the exception of one comparison (> Bachelor’s degree vs. some college). Respondents with self-rated health of fair, good, very good, and excellent more often reported frequent handwashing than those with self-rated poor health. Respondents with good and very good self-rated health more often reported frequent handwashing than those with fair self-rated health. Respondents living in the northeast region of the U.S. more often reported frequent handwashing than those living in the south and west. No differences by race/ethnicity or metropolitan status were significant.

#### Disinfecting (Tables 1 and 2)

Nearly three-fourths (74.0%) of respondents said they were disinfecting surfaces at home and work often to prevent COVID-19. Women more often than men reported frequent surface disinfection. Respondents aged 45–59 years old more often reported frequent surface disinfection than those aged 18–29 and 30–44 years old. Respondents 60 years of age and older more often reported frequent surface disinfection than those aged 18–29 years old. Respondents in the higher income categories ($25k–$49k, 50k–$99k, and ≥ $100k) more often reported frequent surface disinfection than those in the lowest category (< $25k). Respondents with some college more often reported frequent disinfection than respondents with a Bachelor’s degree or more education. Non-Hispanic Blacks and Hispanics, compared to non-Hispanic Whites and those identifying as Multiracial or other, more often reported frequent surface disinfection. Respondents with self-rated health of very good and excellent more often reported frequent surface disinfection than respondents with poor self-rated health. Those with good, very good, and excellent, compared to fair, self-rated health more often reported frequent surface disinfection. Respondents living in metropolitan, compared to non-metropolitan, areas more often reported frequent surface disinfection. Those living in the northeast more often reported disinfecting surfaces than those living in the west. No differences by education were significant.

### Discussion

These data from a nationally representative sample suggest that in the spring of 2020, most U.S. adults were taking hygiene-related precautions to prevent COVID-19. The fact that almost all adults surveyed said that they were washing their hands often with soap and water to prevent COVID-19 is encouraging, as handwashing is an important way to prevent the spread of COVID-19 and other viruses.

Nearly three-quarters of adults said that they were disinfecting surfaces at home and work often to prevent COVID-19. This percentage, although high, is not as high as the percentage for handwashing. Compared to handwashing, disinfecting surfaces often may be a relatively novel behavior (i.e., people may be less familiar with the importance of frequent disinfection than they are with frequent handwashing) potentially making it harder to adopt this practice [6]. Disinfecting surfaces frequently could be more time-consuming and difficult than handwashing; this could contribute to the lower adherence to this practice. Unfamiliarity with how to disinfect surfaces [7] and, possibly, unavailability and cost of disinfectant, may also contribute to this lower adherence percentage.

This study identified several demographic characteristics associated with hygiene-related practices. Men less often reported both handwashing and disinfecting; this finding is consistent with previous research showing that men engage in protective behaviors (e.g., handwashing) less often than women, even during epidemics [8, 9], and a finding consistent with work conducted during the COVID-19 pandemic [10]. Additionally, those with self-rated poor health were less likely to report handwashing and disinfecting to prevent COVID-19. As evidence indicates that COVID-19 disproportionately affects men and people with underlying health conditions, it may be particularly important for men and people in poor health to take preventive action to protect themselves from COVID-19 [11, 12].

Additional demographic characteristics were associated with a higher prevalence of hygiene-related COVID-19 prevention practices. Generally, frequent handwashing and disinfecting increased with age, although the pattern was stronger for handwashing. Compared to non-Hispanic Whites and those identifying as Multiracial or other, non-Hispanic Blacks and Hispanics reported higher disinfecting rates. Current data indicate that COVID-19 causes a disproportionate burden of severe illness and death among older people and among racial and ethnic minority groups [11–13]. It is unclear why these particular groups had higher rates of prevention practices, while other vulnerable groups (i.e., men and people in poor health) did not. More research is needed to clarify these relationships. These data, along with data showing a high rate of cloth face covering use among non-Hispanic Blacks and Hispanics [14], also suggest that individual adherence to CDC guidance is unlikely to explain the racial/ethnic disparity in rates and outcomes of COVID-19; rather, this may be due to systemic racial inequity issues.

Our findings also suggest that people with lower income and education levels are less likely to take these precautions, although these patterns were, again, stronger for handwashing. People with lower income or education levels may face barriers to engaging in these behaviors. For example, they may be more likely to be classified as essential workers and work outside the home during the pandemic [15] and therefore have less time to clean. They may also have less access to water to use for handwashing; recent data indicate that lower income communities disproportionately lack access to clean water and indoor plumbing [16]. More research is needed to better understand the relationship between income, education, and these hygiene practices.

Where people live seems to be related to hygiene-related prevention practices. Those in the south and west regions of the U.S. had lower rates of handwashing compared to those living in the northeast and people living in non-metropolitan areas had lower rates of disinfecting surfaces than those living in metropolitan areas. These findings may be related to the fact that when the survey was conducted, most COVID-19 cases were in more densely populated metropolitan areas and a large COVID-19 outbreak was occurring in the northeast region of the U.S. [17].

## Conclusions

During March 19–April 9, 2020, most survey respondents reported frequent handwashing and surface disinfection to prevent COVID-19. These practices are important components of a layered approach to COVID-19 prevention, which also includes wearing masks and physical distancing. We also identified gaps in these self-reported practices; 7% of respondents said they were not handwashing often and 26% said they were not disinfecting surfaces often. We also found that men, younger people, people with lower incomes and lower education levels, and people in self-rated poor health generally reported lower rates of both frequent handwashing and surface disinfection. These practice gaps among sub-populations vulnerable to COVID-19 (men and people in poor health) [11, 12] are of particular concern. Practice gaps for those with low incomes are also concerning, given that underlying health conditions are more prevalent in this group [18]. Communication efforts could be informed and enhanced by research delineating why some sub-populations have lower rates of hygiene-related COVID-19 prevention practices. Additionally, more work is needed to understand how hygiene behaviors fit in with other recommended COVID-19 prevention behaviors, such as wearing masks and physical distancing.

## Limitations

- Although the data were weighted to be nationally representative and computers and internet access were provided to respondents who needed them, the results may not be fully representative of the US adult population (e.g., those uncomfortable with the internet may have had lower participation).
- The data might be affected by social desirability bias, resulting in an overreporting of socially desirable behaviors (e.g., handwashing).
- These data only measure self-reported practices, not actual practices.
- Many of the demographic characteristics covary, which may confound results.
- These data do not allow causal inferences about the reasons for differences by demographic characteristics found in self-reported practices.

## Data Availability

The dataset supporting the conclusions of this article can be licensed from Porter Novelli Public Services by contacting Deanne Weber (deanne.weber@porternovelli.com).

## Abbreviations

CDC: Centers for Disease Control and Prevention
CI: Confidence interval
COVID-19: Coronavirus disease 2019
PR: Prevalence ratio
